# Sex Differences in Hypothalamic Changes and the Metabolic Response of TgAPP Mice to a High Fat Diet

**DOI:** 10.3389/fnana.2022.910477

**Published:** 2022-07-26

**Authors:** Alejandra Freire-Regatillo, Sonia Diaz-Pacheco, Laura M. Frago, María-Ángeles Arévalo, Jesús Argente, Luis M. Garcia-Segura, María L. de Ceballos, Julie A. Chowen

**Affiliations:** ^1^Department of Endocrinology, Instituto de Investigación la Princesa, Hospital Infantil Universitario Niño Jesús, Madrid, Spain; ^2^Department of Pediatrics, Universidad Aútonoma de Madrid, Madrid, Spain; ^3^Cajal Institute, CSIC, Madrid, Spain; ^4^Centre for Biomedical Network Research for Physiopathology of Obesity and Nutrition (CIBEROBN), Instituto de Salud Carlos III, Madrid, Spain; ^5^Centre for Biomedical Network Research for Frailty and Healthy Ageing (CIBERFES), Instituto de Salud Carlos III, Madrid, Spain; ^6^IMDEA Food Institute, CEI UAM + CSIC, Madrid, Spain

**Keywords:** aging, amyloid precursor protein, diet, hypothalamus, inflammation, metabolism, sex differences

## Abstract

The propensity to develop neurodegenerative diseases is influenced by diverse factors including genetic background, sex, lifestyle, including dietary habits and being overweight, and age. Indeed, with aging, there is an increased incidence of obesity and neurodegenerative processes, both of which are associated with inflammatory responses, in a sex-specific manner. High fat diet (HFD) commonly leads to obesity and markedly affects metabolism, both peripherally and centrally. Here we analyzed the metabolic and inflammatory responses of middle-aged (11–12 months old) transgenic amyloid precursor protein (TgAPP) mice of both sexes to HFD for 18 weeks (starting at 7–8 months of age). We found clear sex differences with females gaining significantly more weight and fat mass than males, with a larger increase in circulating leptin levels and expression of inflammatory markers in visceral adipose tissue. Glycemia and insulin levels increased in HFD fed mice of both sexes, with TgAPP mice being more affected than wild type (WT) mice. In the hypothalamus, murine amyloid β (Aβ) levels were increased by HFD intake exclusively in males, reaching statistical significance in TgAPP males. On a low fat diet (LFD), TgAPP males had significantly lower mRNA levels of the anorexigenic neuropeptide proopiomelanocortin (POMC) than WT males, with HFD intake decreasing the expression of the orexigenic neuropeptides Agouti-related peptide (AgRP) and neuropeptide Y (NPY), especially in TgAPP mice. In females, HFD increased POMC mRNA levels but had no effect on AgRP or NPY mRNA levels, and with no effect on genotype. There was no effect of diet or genotype on the hypothalamic inflammatory markers analyzed or the astrogliosis marker glial acidic protein (GFAP); however, levels of the microglial marker Iba-1 increased selectively in male TgAPP mice. In summary, the response to HFD intake was significantly affected by sex, with fewer effects due to genotype. Hypothalamic inflammatory cytokine expression and astrogliosis were little affected by HFD in middle-aged mice, although in TgAPP males, which showed increased Aβ, there was microglial activation. Thus, excess intake of diets high in fat should be avoided because of its possible detrimental consequences.

## Introduction

Obesity is one of the most important health problems facing developed countries not only because it has reached epidemic proportions, but also because this disease increases the risk for important comorbidities and mortality (Malik et al., 2013; Sarma et al., 2021). The obesity epidemic is the result of deleterious changes in lifestyle, including both decreased physical activity and poor dietary habits (e.g., excess consumption of a high carbohydrate/high fat diet). The relationship between obesity and insulin resistance/type 2 diabetes and cardiovascular complications is widely accepted, but the detrimental health effects of obesity are more comprehensive. Moreover, men and women differ in their propensity to become obese, as well as in their development of obesity-associated health complications, which is partially due to differences in sex steroid levels (Chowen et al., 2019).

Alzheimer’s disease (AD), which is characterized by the deposition of β-amyloid peptide (Aβ) in senile plaques, neurofibrillary tangles composed of hyperphosphorylated tau, loss of specific subsets of neurons, and neuroinflammation resulting from astrogliosis and microglial activation, is the main cause of dementia. The cardinal symptoms of this neurologic disease are progressive learning and memory deterioration and although advancing age is the most significant risk factor for the development of neurodegenerative processes such as AD (Guerreiro and Bras, 2015), other factors contribute to its onset (Moser and Pike, 2016; Sala Frigerio et al., 2019). Indeed, the incidence of AD is higher in women compared to men (Carr et al., 1997; Fisher et al., 2018), a difference that likely results from differences in sex steroid effects (Li and Singh, 2014; Moser and Pike, 2016). Likewise, in transgenic (Tg) models of AD female mice are reported to show earlier Aβ deposition and cognitive deficits than males (Callahan et al., 2001; Yang et al., 2018).

Another important risk factor for developing AD is obesity. Indeed, a 27-year longitudinal population-based study indicates that midlife obesity raises the risk of future dementia (Whitmer et al., 2005), and accumulating evidence suggests that cognitive dysfunction should be prominent on the list of the adverse health consequences of living in an obesogenic environment (Smith et al., 2011; Martin and Davidson, 2014). Excess HFD consumption usually leads to obesity. The effects of HFD on the hippocampus, a key center for cognition, have been studied in rodents where it has been shown to increase inflammatory markers and induce neuroinflammation, as well as neuron loss and plaque formation (Kim et al., 2016). Importantly, these changes were associated with marked deterioration of memory processes including spatial navigation, which is hippocampal-dependent. In different transgenic models of AD, HFD intake has been shown to exacerbate memory deficits and hippocampal inflammatory markers and this is associated with glial activation (Jayaraman et al., 2014; Tucsek et al., 2014). In some studies, HFD was also reported to increase Aβ deposition in AD transgenic mice (Cao et al., 2007; Maesako et al., 2012), but in others, no changes were observed (Knight et al., 2014) and interestingly, sex differences in these processes have been reported.

The symptoms of AD are not restricted to cognitive alterations. Indeed, metabolic abnormalities, such as changes in body weight, and neuroendocrine functions are also present and often precede the detection of cognitive decline (for review see Ishii and Iadecola, 2015). In recent years AD has been increasingly viewed as a metabolic disorder, exhibiting impaired brain responses to glucose and insulin, with increased insulin and insulin-like growth factor (IGF) resistance (Talbot et al., 2012; Ishii et al., 2014). The hypothalamus has a prominent role in integrating peripheral nutritional, metabolic, and hormonal signals to control whole-body energy homeostasis. By employing imaging techniques, abnormalities in this brain region, including decreased hypothalamic volume (Loskutova et al., 2010) and atrophy (Callen et al., 2001), have been described in AD patients. Moreover, both senile plaques and neurofibrillary tangles have been demonstrated in the hypothalamus of AD subjects (Ogomori et al., 1989; Braak and Braak, 1991), albeit to less extent compared to what is described in the cerebral cortex or hippocampus. Different hypothalamic neuropeptides involved in metabolic control are also reported to be altered and although glycemia and circulating insulin levels are usually unaffected, insulin resistance is a common feature of AD with reduced signaling at central insulin and IGF-1 receptors (Steen et al., 2005; Talbot et al., 2012), contributing to deficits in neural function, synaptic plasticity, and cellular integrity. Moreover, plasma leptin levels and hippocampal leptin signaling are reduced in AD (Power et al., 2001; Lieb et al., 2009), resulting in a state of central leptin resistance. There is also evidence of hypothalamic alterations and metabolic dysfunction in rodent models of AD and following HFD. Ishii and Iadecola (2015) reported low plasma leptin levels in Tg2576 mice with low body weight at 3 months of age, before amyloid plaque formation, with the absence of hyperpolarizing responses to leptin in the hypothalamic neuropeptide Y (NPY). In TgAPP/PS1 mice a hypermetabolic state was identified at 5 months of age, by means of NMR spectrometry, and this change subsided at 10 months of age (Zheng et al., 2018). Others have shown that HFD worsens weight gain, glucose intolerance, changes in circulating glucose and insulin, and adiposity in AD transgenics (Ho et al., 2004; Barron et al., 2013; Ruiz et al., 2016; Robison et al., 2020).

As mentioned above, many processes associated to AD are sex-dependent, such as the metabolic and weight gain induced by an HFD, the inflammatory response, or glial activation. However, these sex differences have not been thoroughly studied, as usually only male mice are studied, or groups of mixed female and male mice have been used, although a recent study (Robison et al., 2020) reported the effects of sex and HFD on the hypothalamus in a model of AD. Here we complement this study by employing a different AD model, as well as studying the outcomes at a more advanced age. Thus, we analyzed the metabolic response of male and female wild type (WT) and transgenic amyloid protein (TgAPP) mice to a long-term HFD, emphasizing the effects on the hypothalamus, which is fundamental for the control of energy homeostasis.

## Materials and Methods

### Animals

This study was designed according to the European Community Council Directive (86/609/EEC; 2010/63/UE) and NIH guidelines for animal care and complied with the Royal Decree 53/2013 pertaining to the protection of experimental animals. The studies and use of animals were approved by the animal care and use committee of the Cajal Institute (Comité de Experimentación Animal del Instituto Cajal) and the Consejeria del Medio Ambiente y Territorio (Comunidad de Madrid, Ref. PROEX 200/14).

The TgAPP mice (line 2576) were raised in our in-house colony (Instituto Cajal, CSIC). These mice were created from a C57BL/6 background by heterozygous breeding of mice expressing the human APP long isoform with two mutations [Lys 670-Asn and Met 671-Leu (Swedish mutation)] under transcriptional control of the hamster prion promoter (Hsiao et al., 1996). WT littermates were used as controls. TgAPP mice and their WT littermates of the same sex were housed together in cages (*n* = 3–5 mice/cage). All mice were maintained at a constant temperature (21 ± 1°C) and humidity (50 ± 1%) in a 12-h light-dark cycle.

They were fed a normal rodent chow diet (3.96 Kcal/g, 5.3% fat, 18.9% protein, 3.9% fiber, soy-free, plus amino acids and vitamins, Rod18-H, Altromin) and given water *ad libitum* until subjected to the experimental diets. The different cages were then randomly separated into two groups receiving either a low-fat diet (LFD; 3.76 Kcal/g, 10.2% kcal from fat, 18% kcal from proteins, 72% kcal from carbohydrates; LabDiet, Sodispan Research SL, Madrid, Spain) or HFD (5.1 Kcal/g, 61.6% kcal from fat, 18%, kcal from proteins, 20% kcal from carbohydrates; LabDiet) for 18 weeks starting at 7–8 months of age. This resulted in eight groups of mice: male and female WTLF (*n* = 5), WTHF (*n* = 5), APPLF (*n* = 9) and APPHF (*n* = 9). Body weight was measured weekly. As WT and APP mice were housed in the same cage, to avoid aggressive behavior, energy intake according to genotype was not calculated but monitored as average energy intake per cage (Yanguas-Casas et al., 2021). Two days before sacrifice the animals were subjected to an overnight fast and glycemia was measured using Glucocard Memory meter strips (Menarini, Spain). All animals were sacrificed by decapitation at the same time of the day (10–14 h). The hypothalamus and subcutaneous, visceral, and brown fat pads were extracted. The fat pads were weighed and frozen in dry ice. Dissection of the brains was performed rapidly on a cold surface before freezing it in dry ice. Trunk blood was collected, allowed to clot, centrifuged (3,000 rpm for 10 min at 4°C) and the serum removed. All tissues were stored at −80°C until processed.

### Serum Analysis

Circulating leptin, insulin, interleukin (IL) 6, tumor necrosis factor α (TNFα), monocyte chemoattractant protein (MCP)-1, and total plasminogen activator inhibitor (PAI)-1 were determined by multiplexed immunoassay according to the manufacturer’s instructions (Millipore, Billerica, MA) in a Bio-Plex suspension array system 200 (Bio-Rad Laboratories, Hercules, CA, USA). Mean fluorescence intensity was analyzed by using Bio-Plex Manager Software 4.1. All samples were run in duplicate and within the same assay for all analyses. The minimum detectable concentrations of IL-6, insulin, leptin, MCO-1, PAI-1, and TNFα were 2.3, 13.0, 4.2, 4.9, 4.0, and 5.3 pg/ml, respectively. The intra-assay coefficients of variation were 5% for all analytes except for TNFα, which was 4%. The inter-assay coefficients of variation of IL-6, insulin, leptin, MCO-1, PAI-1, and TNFα were 11, 11, 10, 10, 15, and 20%, respectively.

Circulating triglycerides (Spinreact S.A., Sant Esteve de Bas, Spain) and non-esterified fatty acids (NEFA; Wako, Neuss, Germany) were measured using enzymatic colorimetric kits according to the manufacturers’ instructions.

### RNA and Protein Isolation

Total mRNA was isolated from brain tissue by using an RNeasy^®^ Plus Mini Kit (Qiagen, Hilden, Germany) and from fatty tissues with the RNeasy^®^ Lipid Tissue Mini Kit (Qiagen). Protein was isolated in both cases by collecting the first elution from the RNeasy^®^ Mini Spin columns and diluting 1:5 with cold acetone. It was then stored at −20°C for at least 30 min before centrifuging (3,000 rpm during 10 min at 25°C), after which the acetone was removed and the pellet resuspended in 100 μl of 3-3-cholamidopropyl dimethylammonio propanesulfonate (CHAPS) buffer [7 mm urea, 2 M thiourea, 4% (w/v) CHAPS, and 0.5% (v/v) 1 M Tris; pH 8.8]. Protein samples were stored at −20°C until protein quantification by the method of Bradford (Bio-Rad Laboratories). Total mRNA samples were stored at −80°C measurement of mRNA purity and concentration using a Nanodrop. Using 1 μg of each RNA sample, cDNA was synthesized with a high-capacity cDNA RT kit (Applied Biosystems, Foster City, CA) and stored at −20°C.

### RT-qPCR

Quantitative RT-PCR was performed by using TaqMan Universal PCR Master Mix (Applied biosystems) and TaqMan Gene Expression Assay-on-demand kits to analyze neuropeptides and receptors involved in metabolic control, including neuropeptide Y (NPY; Mm03048253_m1), Agouti-related protein (AgRP; Mm00475829_g1), proopiomelanocortin (POMC; Mm00435874_m1), IL6 (Mm00446190_m1), IL1-β (Mm01336189_m1), TNFα (Mm00443260_g1), DNA-damage inducible transcript 3 (DDIT3; Mm01135937_g1). All samples were run in duplicate. Various housekeeping genes were tested and those that did not vary between experimental groups were chosen to normalize the data [phosphoglycerate kinase 1 (Pgk1; Mm00435617_m1) and beta actin (Actb; Mm00607939_s1) in hypothalamic samples and ribosomal RNA 18S (Mm03928990_g1) and Actb for adipose tissue]. The ΔΔCT method was used to determine relative expression levels and for statistical analysis. All data are expressed as % WT male LFD.

### Western Blotting

For Western blotting, 10 μg of protein was resolved in a 15% SDS-acrylamide gel under denaturing conditions. Proteins were then transferred to a polyvinylidine difluoride membrane (Bio-Rad, Hercules, CA, USA). Transfer efficiency was determined by Ponceau red dyeing. Membranes were blocked with Tris-buffered saline (TBS) containing 5% (w/v) non-fat milk or bovine serum albumin (for phosphorylated proteins) and incubated with the appropriate primary antibody and concentration overnight at 4°C under agitation. The antibodies used were anti-GFAP (Sigma-Aldrich, Darmstadt, Germany), anti-ionized calcium-binding adapter molecule (Iba)-1 (Wako Chemicals, Richmond VA, USA), anti-vimentin (Sigma-Aldrich), anti-c-jun N terminal kinase (JNK, Santa Cruz Biotechnology, Santa Cruz, CA, USA), anti-phosphorylated- JNK (pJNK, Promega. Madison, WI, USA), anti-pNFκB (Cell Signaling, Danvers, MA, USA), anti-NFκB (ThermoFisher, Waltham, MA, USA), and anti-pIκBa (Cell Signaling).

The following day the membranes were washed and incubated with the secondary antibody conjugated with peroxidase (Pierce Biotechnology, 1:1,000 or 1:2,000). Peroxidase activity was visualized by using chemiluminescence and quantified by densitometry using an Image-Quant LAS4000 mini–TL Software (GE Healthcare Europe GmbH, Spain). All blots were rehybridized with actin to adjust for loading and then normalized to % male LFD values on each gel.

### Levels of β-Amyloid in the Hypothalamus

Amyloid-β peptide-42 detection in the hypothalamus was performed using a mouse Aß42 ELISA kit (Invitrogen, Carlsbad, CA, USA) on hypothalamic protein samples diluted 1:10, following the manufacturer’s instructions.

### Statistical Analysis

The programs SPSS version 19.0 (SPSS Inc., Chicago, IL, USA) and GraphPad Prism 8 (GraphPad Software, Inc., San Diego, CA, USA) were used for data analysis. A three-way ANOVA was performed (factors: genotype, diet, and sex), followed by two-way and one-way ANOVAs if appropriate. Scheffé’s f test was used as a post-hoc test to determine whether specific differences existed between the experimental groups. If data were non-parametric, a Kruskal-Wallis test with Dunn’s pair test was performed. All data are presented as mean ± SEM. The results were considered statistically significant at *p* < 0.05. The *p*-values in the figures represent the results of the one-way ANOVA, the Student’s t-test after factor separation, or the Kruskal-Wallis test.

## Results

### Weight, Weight Gain, and Fat Mass

**Figure 1:**
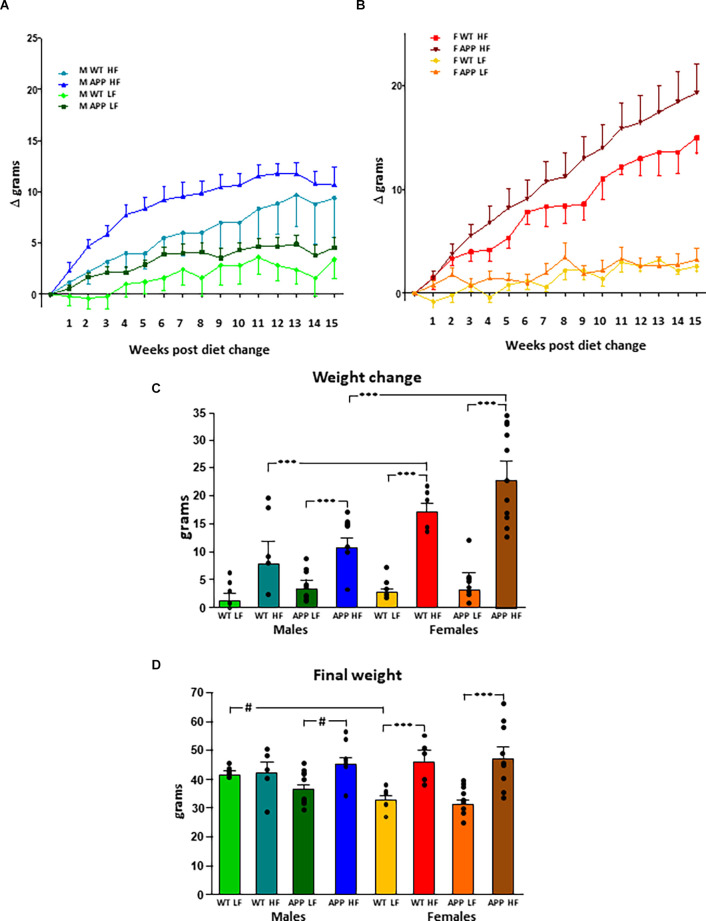
Modifications in body weight over time in male **(A)** and female **(B)** mice after being subjected to a low fat (LF) or high fat (HF) diet. The total accumulated change in body weight throughout the study **(C)** and the final body weight **(D)**. WT, wild type; APP, transgenic for amyloid precursor protein. Bar graphs represent the mean ± SEM of five mice per experimental group for the WT mice and nine mice per experimental group for the TgAPP mice. ^***^ = *p* < 0.0001; # = *p* < 0.02.

The results of the statistical analysis of the final weight, weight gain, and fat mass can be found in Table 1. At the onset of the study, males weighed more than females, but there was no effect of genotype in either sex. Although the weights throughout the study were analyzed in both sexes together, for clearer representation these data are shown separated by sex (Figures 1A,B). There was an effect of sex and diet on weight gain over time, with interactions between sex and diet and sex and genotype. Total weight gain throughout the study (Figure 1C) was determined by all three factors, with interactions between sex and diet, genotype and, diet and sex, genotype, and diet. In males, HFD intake increased weight gain in both genotypes but it only reached statistical significance in APP mice. Male APP mice also gained more weight on the LFD than WT males, suggesting an increased susceptibility to weight gain on different diets. All females on the HFD gained significantly more weight than those on the LFD and, although APP females tended to gain more weight than WT on the HFD, this was not significant. There was a clear sex difference in the response to the HFD with both WT and APP females gaining more weight on this diet than their male counterparts.

**Table 1:** Significant results of the three-way ANOVA, showing the value of F (degrees of freedom between groups, degrees of freedom within groups) and the *p*-value of the weight change, final weight, and the percentage of visceral adipose tissue (% VAT), subcutaneous adipose tissue (% SCAT), and % brown adipose tissue (% BAT).

	**Interaction**			**Independent effect**		
	**Factors**	**F**	***p*-value**	**Factor**	**F**	***p*-value**	
Weight Gain over time	Sex-Diet	*F*_(19,893)_ = 6.6	*p* < 0.0001	Sex	*F*_(19,893)_ = 8.7	*p* < 0.0001
	Sex-Genotype	*F*_(19,893)_ = 1.7	*p* < 0.03	Diet	*F*_(19,893)_ = 26.7	*p* < 0.0001
Total Weight Gain	Sex-Diet	*F*_(11,114)_ = 26.5	*p* < 0.0001	Sex	*F*_(11,114)_ = 234.5	*p* < 0.0001
	Diet-Genotype	*F*_(11,114)_ = 47.3	*p* < 0.0001	Diet	*F*_(11,114)_ = 180.6	*p* < 0.0001
	Sex-Diet-Genotype	*F*_(11,114)_ = 23.2	*p* < 0.0001	Genotype	*F*_(11,114)_ = 3.9	*p* < 0.05
Final Body Weight	Sex-Diet	*F*_(1,48)_ = 7.3	*p* < 0.01	Diet	*F*_(1,48)_ = 22.6	*p* < 0.0001
% VAT	Sex-Diet	*F*_(1,55)_ = 19.4	*p* < 0.0001	Sex	*F*_(1,55)_ = 71.6	*p* < 0.0001
				Diet	*F*_(1,55)_ = 29.6	*p* < 0.004
% SCAT	Diet-Genotype	*F*_(1,48)_ = 4.1	*p* < 0.05	Sex	*F*_(1,48)_ = 72.8	*p* < 0.0001
				Diet	*F*_(1,48)_ = 63.6	*p* < 0.0001
% BAT		NS		Sex	*F*_(1,48)_ = 12.1	*p* < 0.001

*NS, not significant*.

Diet was a determining factor in the final body weight (Figure 1D), with an interaction between sex and diet. In males, HFD only increased the final body weight in APP mice. However, in females, HFD increased body weight regardless of genotype. Males weighed more than females on the LFD, although this only reached statistical significance in WT mice. However, when on an HFD, as females gained more weight than males, there was no difference between the sexes in body weight at the end of the study.

**Figure 2:**
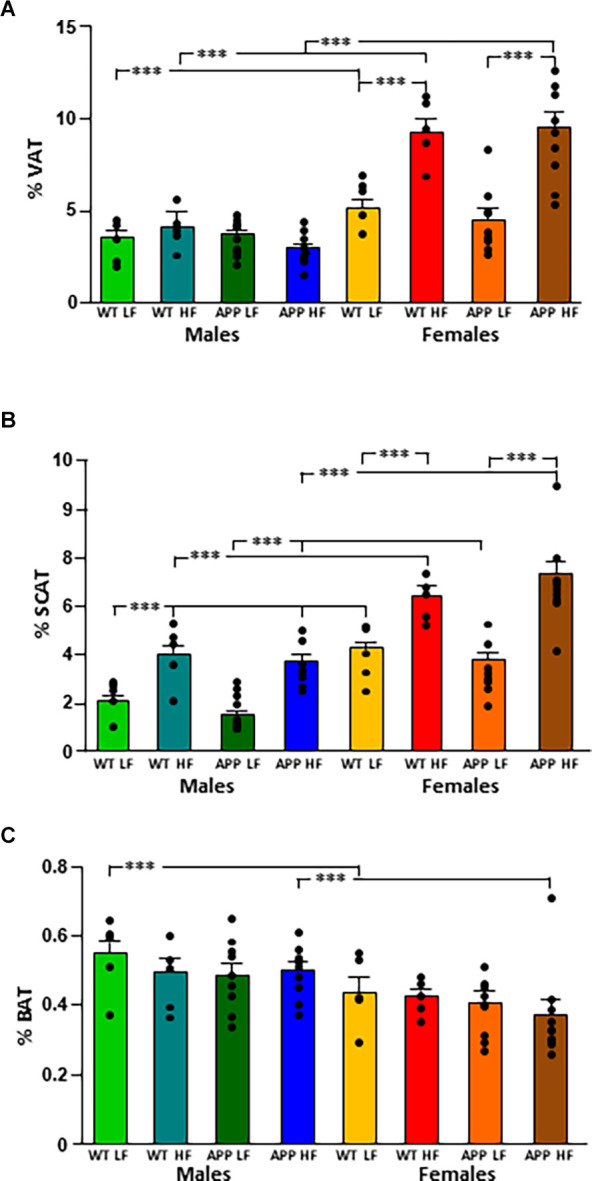
Changes in body composition as depicted by the percent of visceral (VAT; **A**), subcutaneous (SCAT; **B**), and brown (BAT; **C**) adipose tissue depots in male and female mice after being exposed to a low fat (LF) or high fat (HF) diet. WT, wild type; APP, transgenic for amyloid precursor protein. Bar graphs represent the mean ± SEM of five mice per experimental group for the WT mice and nine mice per experimental group for the TgAPP mice. ^***^ = *p* < 0.0001.

The % VAT (Figure 2A) was influenced by sex and diet, with an interaction between these two factors. Indeed, WT females had a higher % VAT than their male counterparts regardless of diet. In contrast in APP mice, females only had significantly higher levels of VAT than their male counterparts when subjected to the HFD. Moreover, while HFD increased the % VAT in both WT and APP females, there was no effect of diet or genotype in males.

Both sex and diet affected the % SCAT (Figure 2B), with an interaction between genotype and diet. Indeed, in male mice, there was an effect of genotype as WTLF mice had a higher % SCAT than male APPLF mice, although this difference was not found when on the HFD. Females of all groups had a higher % SCAT than their corresponding male counterparts and HFD increased the % SCAT in all groups of both sexes.

Male mice had a higher % BAT than females, with this reaching statistical significance in WT mice on the LFD and APP mice on the HFD (Figure 2C). There was no effect of genotype or diet on the percentage of BAT in either sex.

### Circulating Hormones and Cytokines

**Figure 3:**
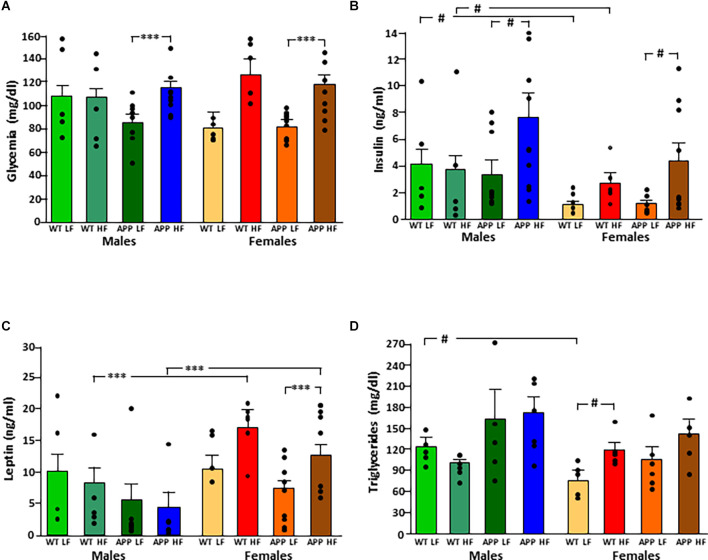
Circulating levels of glucose **(A)**, insulin **(B)**, leptin **(C)**, and triglycerides **(D)** in male and female mice after being exposed to a low fat (LF) or high fat (HF) diet. WT, wild type; APP, transgenic for amyloid precursor protein. Bar graphs represent the mean ± SEM of five mice per experimental group for the WT mice and six mice per experimental group for the TgAPP mice. ^***^ = *p* < 0.0001; # = *p* < 0.02.

The significant results of the statistical analysis of circulating metabolic factors and cytokines are shown in Table 2. The intake of HFD increased glycemia levels in all groups (Figure 3A), although this only reached statistical significance in APP mice of both sexes.

**Table 2:** Significant results of the three-way ANOVA, showing the value of F (degrees of freedom between groups, degrees of freedom within groups) and the *p*-value of circulating metabolic factors and cytokines.

	**Interaction**			**Independent effect**		
	**Factors**	**F**	***p*-value**	**Factor**	**F**	***p*-value**	
Glycemia		NS		Diet	*F*_(1,28)_ = 20.0	*p* < 0.0001
Insulin	Diet-Genotype	*F*_(1,36)_ = 6.1	*p* < 0.02	Sex	*F*_(1,36)_ = 6.8	*p* < 0.02
Leptin	Sex-Diet	*F*_(1,46)_ = 8.5	*p* < 0.005	Sex	*F*_(1,46)_ = 19.4	*p* < 0.0001
Triglycerides	Genotype	*F*_(1,31)_ = 6.0	*p* < 0.02		NS	

*NS, not significant*.

Circulating insulin levels were determined by sex, with an interaction between genotype and diet (Figure 3B). WT females had lower insulin levels than WT males, regardless of diet, but this sex difference was not significant in APP mice. The APP genotype affected the glucose metabolism response to HFD intake as, although circulating insulin levels rose in response to HFD in both genotypes, this increase was only significant in APP mice, and this occurred in both sexes.

Leptin levels were affected by sex, with an interaction between sex and diet. Females had overall higher levels than males, and this was significant in mice on the HFD regardless of genotype (Figure 3C). There was no significant effect of diet in males, while in females HFD induced higher levels of leptin and although this same tendency occurred in both genotypes, it only reached significance in APP females.

Serum triglyceride levels were genotype-dependent with APP mice having overall higher triglyceride levels than their WT counterparts (Figure 3D). In females, HFD significantly increased triglyceride level reaching significance only in WT mice. There was no effect of any factor on circulating NEFA, MCP1, or total PAI1 levels (Table 3). Circulating IL6 and TNFα levels were below the level of detection in samples of all experimental groups.

**Table 3:** Circulating levels of monocyte chemoattractant protein-1 (MCP-1, pg/ml), plasminogen activator inhibitor-1 (PAI-1, pg/ml), and non-esterified fatty acids (NEFA, mM).

	MCP-1	PAI-1	NEFA	
M WT LFD	28.4 ± 6.8	5,713.0 ± 2,350.0	1.24 ± 0.15
M WT HFD	17.2 ± 4.2	9,940.7 ± 4,717.9	0.88 ± 0.08
M APP LFD	27.1 ± 6.1	9,223.3 ± 3,681.6	1.20 ± 0.13
M APP HFD	16.2 ± 3.4	9,152.3 ± 2,917.1	1.12 ± 0.13
F WT LFD	14.0 ± 6.0	1,830.2 ± 371.7	1.15 ± 0.03
F WT HFD	24.2 ± 3.2	3,972.4 ± 1,051.5	1.34 ± 0.13
F APP LFD	21.8 ± 4.8	2,864.1 ± 516.1	1.19 ± 0.16
F APP HFD	14.5 ± 2.6	5,988.7 ± 2,790.9	1.26 ± 0.05

*There were no significant differences between groups. M, male; F, female; WT, wild type; APP, transgenic for amyloid precursor protein; LFD, low fat diet; HFD, high fat diet*.

### Expression of Cytokines in Adipose Tissue

#### Visceral Adipose Tissue

**Figure 4:**
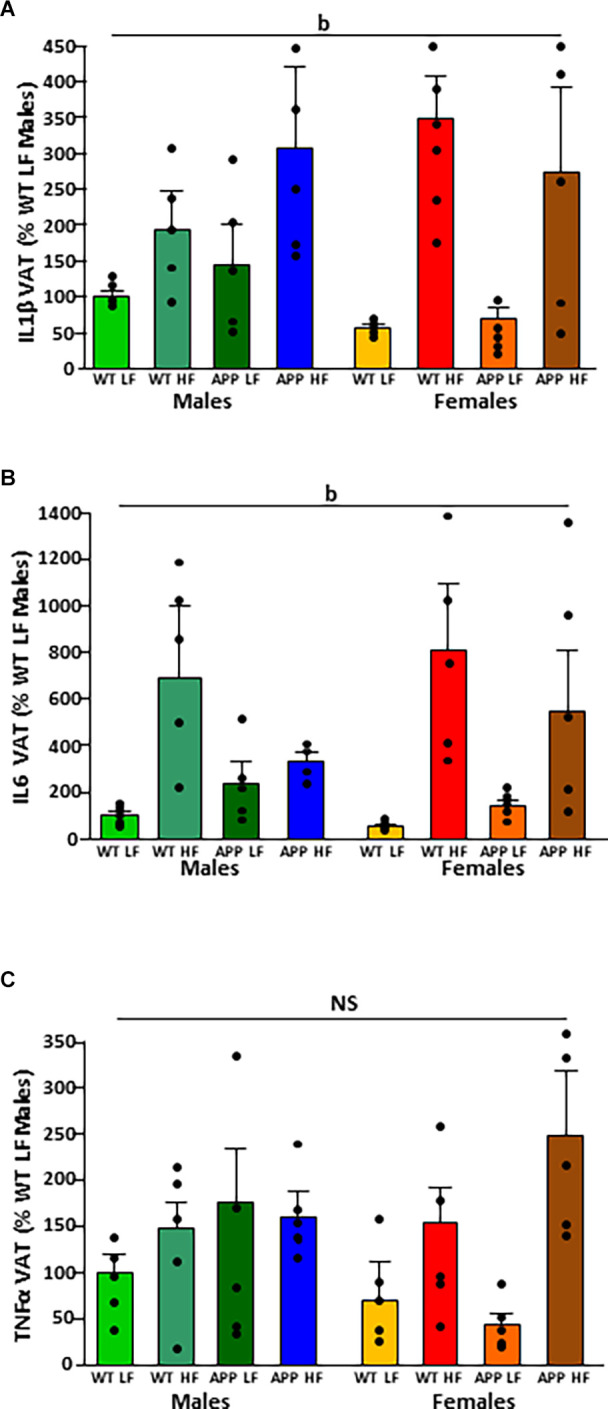
Relative mRNA levels of interleukin (IL)1β **(A)**, IL6 **(B)**, and tumor necrosis factor (TNF)α **(C)** in visceral adipose tissue (VAT) in male and female mice after being exposed to a low fat (LF) or high fat (HF) diet. WT, wild type; APP, transgenic for amyloid precursor protein. Bar graphs represent the mean ± SEM of five mice per experimental group. b = overall effect of diet; NS, non-significant.

The significant results of the analysis of cytokines in adipose tissue are shown in Table 4. The intake of HFD induced the expression of IL1β and IL6 mRNA in VAT (Figures 4A,B, respectively). There were no statistically significant effects of any of the factors analyzed on TNFα mRNA levels (Figure 4C).

**Table 4:** Significant results of the three-way ANOVA, showing the value of F (degrees of freedom between groups, degrees of freedom within groups) and the *p*-value of the analysis of inflammatory factors in visceral adipose tissue (VAT) and subcutaneous adipose tissue (SCAT).

	**Interaction**			**Independent effect**		
	**Factors**	**F**	***p*-value**	**Factor**	**F**	***p*-value**	
VAT IL1β		NS		Diet	*F*_(1,29)_ = 6.3	*p* < 0.02
VAT IL6		NS		Diet	*F*_(1,31)_ = 16.6	*p* < 0.0003
VAT TNFα		NS		NS	
SCAT IL1β		NS		NS	
SCAT IL6		NS		Sex	*F*_(1,36)_ = 5.7	*p* < 0.03
				Diet	*F*_(1,36)_ = 5.4	*p* < 0.03
SCAT TNFα	Sex-Diet	*F*_(1,34)_ = 6.2	*p* < 0.02		NS	

*IL, interleukin; TNF, Tumor necrosis factor; NS, Not significant*.

#### Subcutaneous Adipose Tissue

**Figure 5:**
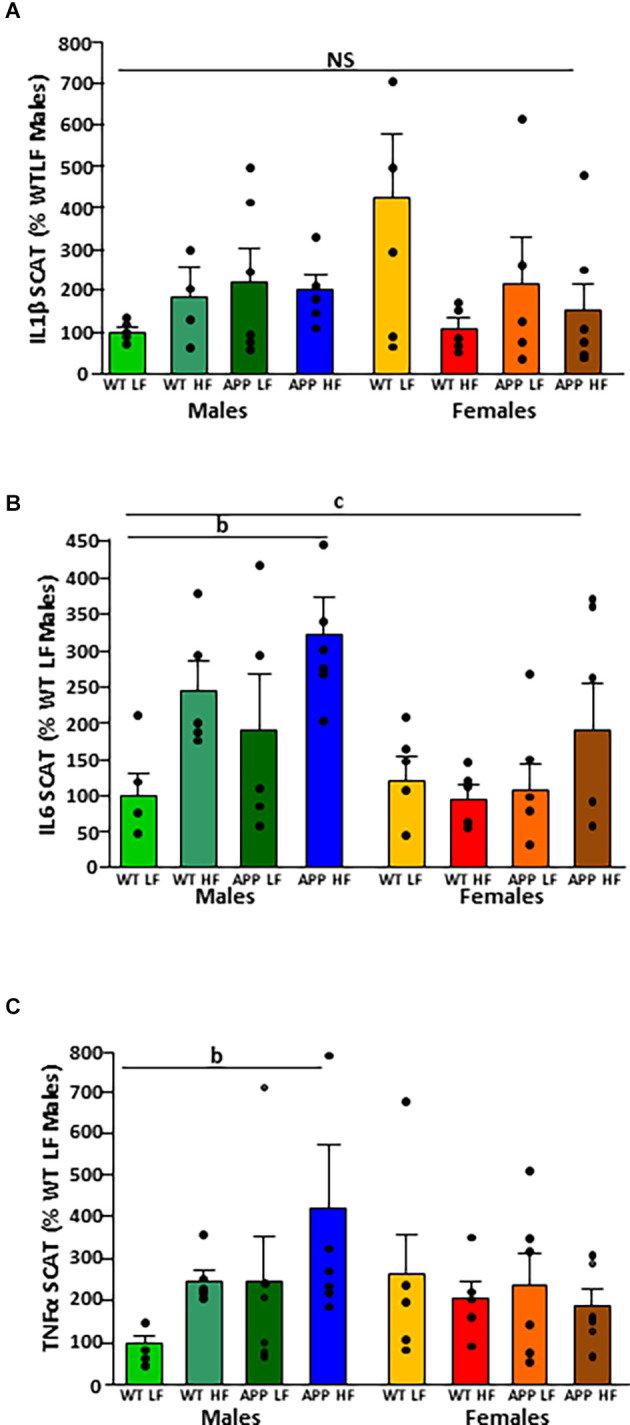
Relative mRNA levels of interleukin (IL)1β **(A)**, IL6 **(B)**, and tumor necrosis factor (TNF)α **(C)** in subcutaneous adipose tissue (SCAT) in male and female mice after being exposed to a low fat (LF) or high fat (HF) diet. WT, wild type; APP, transgenic for amyloid precursor protein. Bar graphs represent the mean ± SEM of five mice per experimental group. b = overall effect of diet, c = overall effect of sex. NS, non-significant.

There was no difference between experimental groups in the expression of IL1β in SCAT (Figure 5A). The mRNA levels of IL-6 in SCAT (Figure 5B) were both sex and diet dependent. Males had overall higher IL6 mRNA levels than females and in males, HFD increased IL6 mRNA levels.

There was an interaction between sex and diet on the mRNA levels of TNFα. There was an overall increase in SCAT TNFα mRNA levels in response to HFD in males (Figure 5C) but with no effect in females.

It is of interest to note that there was no effect of genotype on the expression of any of the cytokines analyzed in VAT or SCAT.

### Modifications in the Hypothalamus

**Figure 6:**
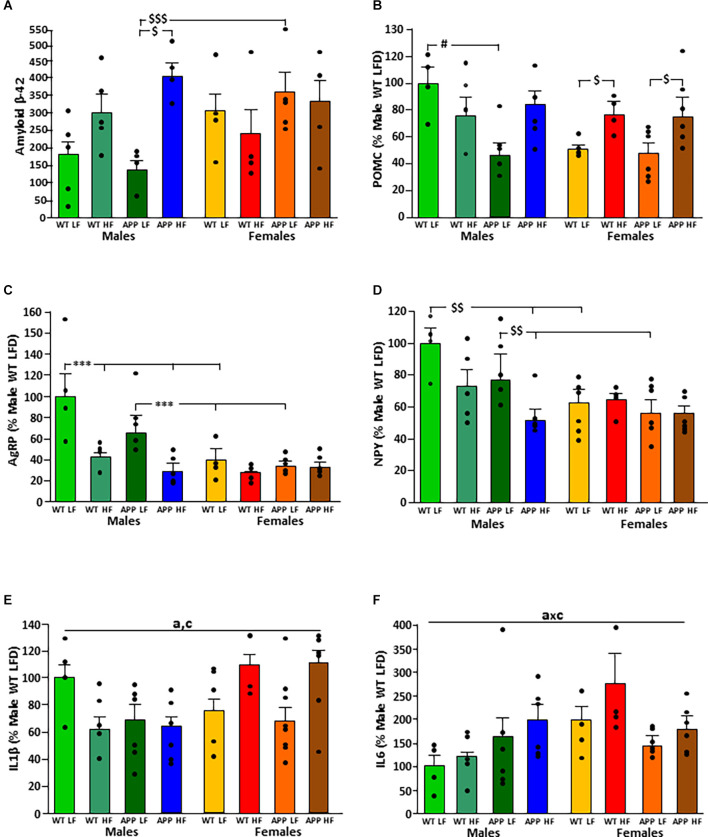
Levels of amyloid β-42 **(A)** and relative mRNA levels of proopiomelanocortin (POMC; **B**), Agouti related peptide (AgRP; **C**), neuropeptide Y (NPY; **D**), interleukin (IL; **E**) 1β and IL6 **(F)** in the hypothalamus of male and female mice after being exposed to a low fat (LF) or high fat (HF) diet. WT, wild type; APP, transgenic for amyloid precursor protein. * = *p* < 0.0001; $ = *p* < 0.05; $$ = *p* < 0.005; $$$ = *p* < 0.0005; # = *p* < 0.02. Bar graphs represent the mean ± SEM of 4–5 mice per experimental group. a, overall effect of genotype; c, overall effect of sex.

The significant results of the statistical analysis of the hypothalamus are shown in Table 5. There was an interaction between the factors of sex and diet on the levels of amyloid-β-42 peptide in the hypothalamus (Figure 6A). When on the LFD males had overall lower levels of amyloid β than females with this reaching significance in APP mice, but on the HFD there were no differences between the sexes. However, HFD only increased the levels of hypothalamic amyloid β in male mice, with this reaching significance in APP males.

**Table 5:** Significant results of the three-way ANOVA, showing the value of F (degrees of freedom between groups, degrees of freedom within groups) and the *p*-value of amyloid beta proopiomelanocortin (POMC), Agouti related peptide (AgRP), neuropeptide Y (NPY), and interleukin (IL)1β in the hypothalamus.

	**Interaction**			**Independent effect**		
	**Factors**	**F**	***p*-value**	**Factor**	**F**	***p*-value**	
Amyloid beta	Sex-Diet	*F*_(1,34)_ = 9.5	*p* < 0.006		NS
Hypothalamic POMC	Sex-Genotype	*F*_(1,30)_ = 4.3	*p* < 0.05	Genotype	*F*_(1,30)_ = 5.9	*p* < 0.02
Hypothalamic AgRP	Sex-Diet	*F*_(1,31)_ = 7.2	*p* < 0.02	Sex	*F*_(1,31)_ = 12.2	*p* < 0.002
				Diet	*F*_(1,31)_ = 12.9	*p* < 0.001
Hypothalamic NPY	Sex-Diet	*F*_(1,33)_ = 4.4	*p* < 0.05	Sex	*F*_(1,33)_ = 4.7	*p* < 0.04
				Genotype	*F*_(1,33)_ = 4.3	*p* < 0.05
Hypothalamic IL1β		NS		Diet	*F*_(1,33)_ = 3.9	= 0.05
				Genotype	*F*_(1, 33)_ = 4.1	= 0.05
Hypothalamic IL6	Sex-Genotype	*F*_(1,33)_ = 5.2	*p* < 0.03		NS	

*NS, not significant*.

The mRNA levels of the anorexigenic neuropeptide POMC (Figure 6B) were dependent on genotype, with an interaction between genotype and sex. In males, APP mice had lower POMC levels than WT mice when on the LFD. In contrast, there was no difference between WT and APP females, with HFD increasing POMC expression regardless of genotype.

Expression of the orexigenic neuropeptide AgRP (Figure 6C) was affected by sex and diet with an interaction between these two factors. When on the LFD, males had higher AgRP mRNA levels than females regardless of genotype. In males, HFD intake reduced AgRP mRNA levels compared to those when on the LFD regardless of genotype. In females, there was no effect of either genotype or diet on AgRP mRNA levels.

The orexigenic neuropeptide NPY (Figure 6D) was affected by genotype and sex, with an interaction between sex and diet. When on the LFD, WT males had higher NPY mRNA levels than WT females and APP males higher than APP females. These differences were not observed when on the HFD. Both WT and APP males on an HFD had lower NPY mRNA levels than those on the LFD, but only reaching significance in APP mice. There was no effect of genotype or diet in females.

Inflammatory markers in the hypothalamus were affected by genotype, sex, and diet. In males, both the APP genotype and HFD intake tended to reduce IL1β mRNA levels, while in females HFD tended to increase the levels of this cytokine regardless of genotype (Figure 6E).

There was an interaction between genotype and sex on the expression of IL6 in the hypothalamus. In WT mice females had overall higher levels than males (Figure 6F). However, there were no significant differences in hypothalamic TNFα mRNA levels or of DDIT3 a marker of ER stress (Table 6). Moreover, no effect of genotype or diet was found on hypothalamic pJNK, pNFκB or pIκBa levels in either sex (Table 7).

**Table 6:** The mRNA levels of tumor nerosis factor (TNF)α and DNA damage-inducible transcript 3 (DDIT3), expressed as a percentage of M WT LFD.

	TNFα	DDIT3
M WT LFD	100 ± 28.3	100 ± 7.6
M WT HFD	88.7 ± 32.3	95.4 ± 10.3
M APP LFD	85.7 ± 35.7	89.6 ± 17.9
M APP HFD	95.9 ± 2.3	95.9 ± 2.3
F WT LFD	80.6 ± 23.8	88.6 ± 5.8
F WT HFD	65.4 ± 13.2	103.1 ± 15.7
F APP LFD	80.2 ± 13.8	103.1 ± 15.7
F APP HFD	83.7 ± 22.0	91.1 ± 3.4

*M, male; F, female; WT, wild type; APP, transgenic for amyloid precursor protein; LFD, low fat diet; HFD, high fat diet*.

### Glial Cell Markers and Inflammatory Signaling in the Hypothalamus

**Figure 7:**
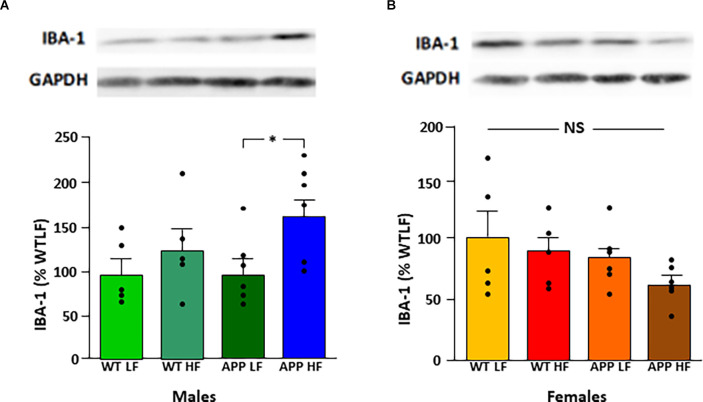
Relative protein levels of ionized calcium binding adaptor molecule 1 (Iba-1) in the hypothalamus of male **(A)** and female **(B)** mice after being exposed to a low fat (LF) or high fat (HF) diet. Bar graphs represent the mean ± SEM 5–6 mice per experimental group. Samples from males and females were analyzed separately and are thus not directly compared. WT, wild type; APP, transgenic for amyloid precursor protein; GAPDH, glyceraldehyde 3-phosphate dehydrogenase; NS, non-significant.

Protein levels of the glial markers GFAP and vimentin were not different between groups in either males or females (Table 7). In contrast, in males HFD significantly increased Iba-1 levels in the hypothalamus of male mice (*F*_(1,18)_ = 5.3, *p* < 0.04), with this effect being significant in APP males (Figure 7), suggesting activation of microglia in males in response to high fat intake.

**Table 7:** Protein levels of phosphorylated jun N terminal kinase (pJNK), pNFκB, pIκBa, glial fibrillary acidic protein (GFAP), and vimentin in the hypothalamus, expressed as a % of the correspondent WT LFD (male or female) group.

	pJNK	pNFκB	pIκBa	GFAP	VIMENTIN
M WT LFD	100,2.6	100,11.4	100,6.2	100,1.2	100,7.6
M WT HFD	107.4,7.7	108.6,16.7	118.4,30.3	105.4,10.1	115.4,18.3
M APP LFD	96.7,6.6	104.2,18.3	72.6,11.8	104.4,11.9	113.6,17.9
M APP HFD	98.8,5.3	91.2,8.8	76.9,8.8	122.9,12.3	121.9,12.3
F WT LFD	100.6,15.6	100,17.8	100,17.5	100,15.8	100,6.6
F WT HFD	83.3,14.7	101.1,15.7	115.7,17.7	113.3,19.7	73.1,19.7
F APP LFD	99.1,17.6	119.8,20.7	108.1,7.3	79.1,15.7	103.1,15.7
F APP HFD	79.4,9.3	116.1,13.4	119.1,23.6	114.1,3.4	98.1,3.4

amyloid precursor protein; LFD, low fat diet; HFD, high fat diet.

## Discussion

Obesity is suggested to be a risk factor for the development of AD and other neurodegenerative diseases (Kivipelto et al., 2005; Pasinetti and Eberstein, 2008; Moser and Pike, 2016). One of the probable causes of this increased risk is the central inflammation associated with poor dietary habits and obesity, which can augment cognitive decline (Smith et al., 2011; Martin and Davidson, 2014). Hypothalamic inflammation and gliosis are also reported to occur in response to chronic HFD intake and weight gain and these processes are associated with the development of metabolic complications, such as insulin resistance, type 2 diabetes, and cardiovascular disease (Horvath et al., 2010; Thaler et al., 2012; Valdearcos et al., 2014, 2015; Robison et al., 2020). The hypothalamus is also affected in AD (Ishii and Iadecola, 2015) and this most likely contributes to the metabolic alterations observed in these patients. The propensity to become obese, develop metabolic diseases, and to suffer neurodegenerative disease increases with age in a sex-specific manner, with central inflammation and gliosis possibly participating in this process (Chowen and Garcia-Segura, 2021). Here we have focused on the metabolic effects of prolonged HFD intake and the alterations in hypothalamic neuropeptides involved in energy balance, with a particular focus on inflammatory markers both in the periphery and hypothalamus, in a model of AD and employing males and females that were middle-aged.

Transgenic mice, harboring different human mutations found in hereditary AD, have been widely used to study the physiopathology of AD (Sasaguri et al., 2022). Indeed, several studies have been conducted in AD transgenic mice subjected to an HFD, and have demonstrated differences in obesity, adiposity, metabolic dysfunction, and alterations in hypothalamic neuropeptides and inflammation. However, few studies have addressed how males and females are differently impacted by an HFD, because only one sex was studied, or groups included both male and female mice. The present work was performed in TgAPP 2576 mice because AD-like symptoms and pathology slowly progress with age, and they show behavioral abnormalities around 12 months of age, even before amyloid plaques develop (Hsiao et al., 1996; Kawarabayashi et al., 2001). There is no neurodegeneration in this transgenic model, in contrast with the human disorder, although glial activation and neuroinflammation, an invariant feature of AD (Heneka et al., 2015), occur. On the other hand, other AD transgenic mice, harboring mutations in APP, presenilin 1 (PS1), and tau, show a rapidly progressing pathology with deposition of Aβ in plaques beginning much earlier, at 4–6 months of age, and with significant learning and memory deficits (Sasaguri et al., 2022). Our mice were 7–8 months of age when the standard diet was switched to an HFD, and they were sacrificed at 11–12 months of age, which is considered middle-aged and approximates 55 years in humans. Furthermore, the mice were group housed with their WT counterparts, to avoid aggressive behavior and maintain a similar housing environment amongst genotypes, since behavioral studies were also conducted on these animals (Yanguas-Casas et al., 2021).

As expected, we found WT male mice to be heavier than WT females at the beginning of the study when all mice were on a normal chow diet. We did not find overt differences in weight or fat mass between male and female TgAPP compared with their WT counterparts, except for a reduction in SCAT in male transgenics, which is in contrast with other authors.

Indeed, in 3xTg-AD mice, a decrease in weight and fat mass was found in males at 7 months of age (Robison et al., 2020), while both male and female 5xFAD mice (6 months of age) showed reductions in body weight, food intake, and energy expenditure (Lopez-Gambero et al., 2021). All groups increased their weight gain when fed an HFD, although this did not reach significance in WT males. Interestingly, female mice gained significantly more weight when subjected to the HFD than males, reaching a similar weight to males ingesting the HFD. This contrasts with what has been reported in many rodent studies, where males are generally more susceptible to the adverse effects of an HFD (Hwang et al., 2010), including AD transgenic mice (Barron et al., 2013). However, our results of increased body weight, visceral and subcutaneous mass in female TgAPP mice, are in essential agreement with the findings of Robison et al. (2020). It is highly probable that the age at which the mice were subjected to the dietary challenge underlies this phenomenon, as the mice used here were middle-aged at the end of the experiment, older than the adult mice that are normally subjected to dietary challenges. Indeed, it has been reported that when employing middle-aged mice, sex differences in diet-induced weight gain and glucose impairment are opposite to that found when using juvenile mice (Nishikawa et al., 2007; Salinero et al., 2018). Likewise, females had a higher percentage of both SCAT and VAT than males, which again is not found at earlier ages. Moreover, both fat pads increased significantly on the HFD in all females, while in males HFD only increased the percentage of SCAT and this was only significant in transgenic mice. Sex differences in fat distribution are well studied in humans, with women tending to accumulate fat in subcutaneous depots, while men store fat in visceral depots (White and Tchoukalova, 2014). Moreover, we found brown adipose tissue to be more abundant in males than in females, which is consistent with previous studies showing that BAT increases at a higher rate than overall body weight in males, but not in females (Valle et al., 2008). As BAT increases metabolic expenditure, this observation could also be involved in the sex differences in metabolic complications during aging.

Circulating metabolic factors were modified by HFD intake in all mice, but the increases in glycemia, insulin, and leptin were significantly increased in the TgAPP mice. These results resemble those of previous studies in TgAPP 2576 female mice (9 months) after 5 months of HFD (Ho et al., 2004), and other transgenics of both sexes (Ho et al., 2004; Kohjima et al., 2010; Freeman et al., 2012), although restricted to male 3xTg-AD mice in another study (Barron et al., 2013). Interestingly, age matters, as 4 weeks of HFD increased plasma glucose and insulin levels in 12-month-old male Tg APP/PS1 mice, while mice of 6 months of age had similar blood glucose levels compared to WT mice, in parallel with glucose intolerance (Ruiz et al., 2016). Triglyceride levels are also reported to be affected by genotype, being higher in TgAPP mice subjected to an HFD (Kohjima et al., 2010; Freeman et al., 2012), although other authors have found unchanged triglyceride levels (Hwang et al., 2010; Ruiz et al., 2016). Together these results indicate that the increased expression of the amyloid precursor protein directly affects systemic metabolism under obesogenic conditions. In contrast, circulating inflammatory factors were not changed in TgAPP mice on the LFD, nor was the expression of inflammatory factors in adipose tissue, although the HFD increased the expression of these factors as previously reported (Tucsek et al., 2014; Kaur and Kaur, 2017). Once again, this lack of change could be due to the age of the animals used in this study, as age itself is also associated with increased inflammatory processes.

The expression of some inflammatory markers has been reported to increase in the hypothalamus of AD mice in some studies. In male 3xTg mice hypothalamic increases in TNF-α and IL-6 were reported (Do et al., 2018). In another study with 5xFAD transgenics, the hypothalamic expression of TNF-α was enhanced in male mice, while that of IL-1β increased in males and females, although IL-1β protein levels were higher only in females (Lopez-Gambero et al., 2021). In these same animals GFAP, an astrocytic marker, and Iba-1, expressed by microglia, were higher in both sexes (Lopez-Gambero et al., 2021). However, we found unaltered expression of pro-inflammatory cytokines and GFAP in our TgAPP mice. Hypothalamic inflammation and gliosis have been reported in response to chronic HFD intake and weight gain and this has been associated with the onset of metabolic complications such as insulin resistance (Horvath et al., 2010; Thaler et al., 2012; Valdearcos et al., 2014; Robison et al., 2020). We found little effect of the transgene on increasing the hypothalamic inflammatory response to HFD intake; indeed, male TgAPP mice had overall lower IL1β mRNA levels. The inflammatory/gliosis response to an HFD differed between the sexes, with females showing increased IL1β mRNA levels, whereas males had increased Iba-1 levels, with this being significant only in TgAPP mice, in agreement with Robison et al. (2020), where no changes in Iba-1 in females was found. However, no other indications of inflammation, ER stress, or astrogliosis, were observed. This is remarkable since in these same animals we reported HFD-induced changes in hippocampal pro-inflammatory and phagocytic-related gene expression, which were more marked in male TgAPP mice (Yanguas-Casas et al., 2021). In contrast, the increased expression of several pro-inflammatory and phagocytic-related genes in female TgAPP on a LFD, returned toward control values in female transgenic mice when subjected to the HFD. It is tempting to argue that higher levels of Aβ in the hippocampus compared to the hypothalamus are responsible for such differences.

Transgenic AD mice have been created by knocking in human genes bearing APP mutations. Therefore, Aβ levels or deposition are assessed with antibodies against the human sequence, with a much greater tendency to aggregate compared to the murine Aβ, and it can only be measured in Tg mice, but not in WT mice. In the present study, we chose to evaluate murine Aβ levels in the hypothalamus, allowing us to quantitate the peptide in all groups under study. Both WT and TgAPP male mice showed increased Aβ in response to HFD intake, reaching significance in the transgenic mice. These results paralleled the increase observed in other studies following prolonged HFD (Kohjima et al., 2010; Barron et al., 2013). Previous studies report no differences in the expression of the enzymes for Aβ synthesis (Tucsek et al., 2014). Because insulin-degrading peptide IDE is known to degrade insulin along with Aβ (Selkoe, 2001; Qiu and Folstein, 2006), in hyperinsulinemia, the binding of insulin to IDE may have interfered with Aβ degradation, leading to Aβ accumulation in the brain. In other words, Aβ blunted degradation would be responsible for increased hypothalamic Aβ levels in HFD male mice. In the case of female mice, insulin levels raised less compared to males, and would not interfere with Aβ degradation, possibly explaining the lack of changes.

The modifications in Iba-1 have a similar pattern to that of hypothalamic Aβ levels, which increase in response to HFD in males, with this increase being significant in TgAPP males. Such an increase is possibly related to the metabolization of circulating fats, as this peptide is involved in lipid processing (Czeczor and McGee, 2017). As neuroinflammation has been associated with aging (Franceschi and Campisi, 2014; Deleidi et al., 2015; Xia et al., 2016), the baseline central inflammation in these middle-aged mice may already be elevated and not significantly changed by the dietary challenge. This possibility deserves further study, as well as morphological analysis of astrocytes and microglia that could shed more light on the affection of these glial cells and their activational state.

Control of energy homeostasis in the hypothalamus resides in the interplay of orexigenic neuropeptides such as NPY and AgRP, to stimulate appetite and decrease energy expenditure with promotes body mass increase, while anorexigenic peptides such as POMC, cause weight loss by inhibiting food intake and stimulating energy expenditure. Modifications in the hypothalamic expression of metabolic neuropeptides in response to HFD were clearly sex-dependent, but they were also affected by genotype in a sex-specific manner. In 3-month-old Tg2576 mice of both sexes, decreased expression in POMC has been reported, while that of NPY and AgRP were unchanged compared to WT mice (Ishii et al., 2014), although decreased AgRP expression was found in 3xTg mice (Do et al., 2018), with no changes in POMC. On an LFD, male TgAPP mice had lower POMC mRNA levels than WT males (Ishii et al., 2014), while on an HFD they had lower levels of NPY mRNA. The orexigenic neuropeptides NPY and AgRPwere both decreased in HFD-fed males, while HFD-fed females increased the expression of the anorexigenic peptide POMC. Interestingly, lower expression of AgRP in male 3xTg mice submitted to an HFD has been reported, along with a decrease in POMC and NPY positive neurons in the hypothalamus (Do et al., 2018). These sex difference in the regulation of metabolic neuropeptides was also found in our previous study (Mela et al., 2012) in peripubertal mice and may be related to the different strategies that males and females follow in response to a dietary challenge.

Although TgAPP mice cannot be directly compared to what occurs in AD, they are considered useful for the study of preclinical AD. Indeed, middle-aged mice, as used here, are too young to develop all signs of AD, but they already show some cognitive decline (Martin-Moreno et al., 2012; Nunez-Borque et al., 2020). Overall, our results suggest that there is an interaction between APP over-expression and energy homeostasis as found in other studies (Puig et al., 2017) since deletion of the APP abrogated many of the changes induced by an HFD; however, this interaction appears to be very complex as both age and sex also influence energy homeostasis. The mechanisms underlying the differences between WT and TgAPP genotypes cannot be deduced from the studies reported here, but these results indicate that alterations in APP expression do affect metabolism and that males appear to be more metabolically affected than females. However, it must also be taken into consideration that some of the parameters studied here are more affected by aging in females, which could mask some of the effects of APP overexpression.

One caveat that must be taken into consideration is that the intake of HFD was compared to that of an LFD; although this diet has been commercialized as a control diet to be used in conjunction with the HFD, it has a higher level of carbohydrates than a normal rodent chow diet. This diet also has some metabolic effects when compared to chow intake (Guerra-Cantera et al., 2021). Another limitation of the study is that it was not designed for a strict metabolic study, where the animals are usually individually caged, allowing the food intake to be singly monitored. Indeed, these same animals were used for behavioral studies (Yanguas-Casas et al., 2021), and littermates were housed in the same cage as mentioned earlier.

It is clear that the interaction between diet, aging, and the development of neurodegenerative diseases, which has only begun to be exposed (Moser and Pike, 2016), deserves further investigation. Furthermore, the differences between males and females in their response to poor dietary intake and their propensity to develop obesity, that is modified during aging, must also be factored into this complex equation. In any case, the avoidance of a diet high in saturated fats is a good choice, independent of sex, age, or health.

## Data Availability Statement

The raw data supporting the conclusions of this article will be made available by the authors, without undue reservation.

## Ethics Statement

The animal study was reviewed and approved by Cajal Institute (Comité de Experimentación Animal del Instituto Cajal) and the Consejeria del Medio Ambiente y Territorio (Comunidad de Madrid, Ref. PROEX 200/14).

## Author Contributions

Conception of the study: JAC, LG-S, MLC, and JA. Funding: JAC, LG-S, M-ÁA, MLC, and JA; Animal handling: AF-R, SD-P, and MLC. Sample processing: AF-R, SD-P, and LMF. Data analysis: JAC, LG-S, MLC, LMF, and M-ÁA. Manuscript redaction: AF-R, MLC, and JAC. All authors contributed to the article and approved the submitted version.

## Funding

The authors are funded by grants from the Spanish Ministry of Science and Innovation (BFU2014-51836-C2-1-R to LG-S and M-ÁA, BFU2014-51836-C2-2-R and BFU2017-82565-C21-R2 to JAC and LMF), Spanish Ministry of Education, Culture and Sports (university training grant PU13/00909 to AF-R), Fondo de Investigación Sanitaria (PI1900166 to JA), Madrid Council S2010/BMD-2349 to MLC; Fondos FEDER and Centro de Investigación Biomédica en Red Fisiopatología de Obesidad y Nutrición (CIBEROBN), and Instituto de Salud Carlos III (JA and JAC).

## Conflict of Interest

The authors declare that the research was conducted in the absence of any commercial or financial relationships that could be construed as a potential conflict of interest.

## Publisher’s Note

All claims expressed in this article are solely those of the authors and do not necessarily represent those of their affiliated organizations, or those of the publisher, the editors and the reviewers. Any product that may be evaluated in this article, or claim that may be made by its manufacturer, is not guaranteed or endorsed by the publisher.
